# Surveillance and sociodemographic risk profiling of human soil-transmitted helminth infections in Gabon, Central Africa

**DOI:** 10.1093/trstmh/trag038

**Published:** 2026-04-29

**Authors:** Huan Zhao, Polydor N Mutombo, Rodrigue Mintsa-Nguema, Dieudonné Nkoghe, Julienne Atsame, Francesca Azzato, Eileen Hor, Jee Hinaut, Maryza Graham, Emily Grahn, Matthew Watts, Constantin Constantinoiu, Richard S Bradbury

**Affiliations:** School of Public Health and Tropical Medicine, College of Medicine and Dentistry, James Cook University, Douglas, QLD 4811, Australia; National Centre for Naturopathic Medicine, Southern Cross University, Lismore, NSW 2480, Australia; Africa Centres for Disease Control and Prevention, Addis Ababa 1000, Ethiopia; Research Institute in Tropical Ecology, National Centre for Scientific and Technological Research, Libreville, BP 13354, Gabon; Programme National de Lutte contre les Maladies Parasitaires, Ministère de la Santé, Libreville, BP 2434, Gabon; Programme National de Lutte contre les Maladies Parasitaires, Ministère de la Santé, Libreville, BP 2434, Gabon; Victorian Infectious Diseases Reference Laboratory, Royal Melbourne Hospital at the Peter Doherty Institute for Infection and Immunity, Melbourne, VIC 3000, Australia; Department of Infectious Diseases, University of Melbourne at the Peter Doherty Institute for infection and Immunity, Melbourne, VIC 3000, Australia; Victorian Infectious Diseases Reference Laboratory, Royal Melbourne Hospital at the Peter Doherty Institute for Infection and Immunity, Melbourne, VIC 3000, Australia; Victorian Infectious Diseases Reference Laboratory, Royal Melbourne Hospital at the Peter Doherty Institute for Infection and Immunity, Melbourne, VIC 3000, Australia; Victorian Infectious Diseases Reference Laboratory, Royal Melbourne Hospital at the Peter Doherty Institute for Infection and Immunity, Melbourne, VIC 3000, Australia; School of Public Health and Tropical Medicine, College of Medicine and Dentistry, James Cook University, Douglas, QLD 4811, Australia; Centre for Infectious Diseases and Microbiology, Institute of Clinical Pathology and Medical Research – New South Wales Health Pathology and Western Sydney Local Health District, Westmead Hospital, Westmead, NSW 2145, Australia; Sydney Institute for Infectious Diseases and Westmead Clinical School, University of Sydney, Sydney, NSW 2050, Australia; School of Veterinary Science, College of Science and Engineering, James Cook University, Douglas, QLD 4811, Australia; School of Public Health and Tropical Medicine, College of Medicine and Dentistry, James Cook University, Douglas, QLD 4811, Australia

**Keywords:** Gabon, helminths, *Necator*, prevalence, real-time PCR, *Strongyloides*

## Abstract

**Background:**

Sub-Saharan Africa bears the highest global burden of soil-transmitted helminth (STH) infections. In Gabon, community-level epidemiological data remain limited and no molecular surveillance of STHs has been conducted to date.

**Methods:**

In July 2023, a community-based cross-sectional survey was conducted in rural and urban areas of Ngounié Province, Gabon. Stool samples from 233 participants were analysed using formalin-ethyl acetate sedimentation microscopy and the Allplex GI-Helminth(I) quantitative polymerase chain reaction (qPCR) assay.

**Results:**

Overall, 70.0% (95% CI 63.6 to 75.8) of participants tested positive for at least one STH by microscopy or qPCR. *Trichuris* spp. was the most prevalent helminth (59.2% [95% CI 52.6 to 65.6]), followed by *Ascaris* spp. (48.1% [95% CI 41.5 to 54.7%]), hookworms (18.5% [95% CI 13.7 to 24.0]) and *Strongyloides* spp. (12.0% [95% CI 8.1 to 16.9]). Microscopy identified *Strongyloides fuelleborni* in 2.7% of samples. qPCR detected *Necator americanus* in 16.7% and *Ancylostoma* spp. in 0.4%. Rural residence was independently associated with *Ascaris* spp. (adjusted odds ratio [AOR] 2.50; p=0.007) and *Trichuris* spp. (AOR 2.45; p=0.008) infections. Male sex (AOR 3.83; p=0.010) and age 18–40 y (AOR 4.90; p=0.011) were associated with *Strongyloides* spp. infection.

**Conclusions:**

STH infections remain endemic in certain Gabonese communities. The findings highlight the need for species-specific surveillance using sensitive molecular diagnostics to inform targeted control strategies.

## Introduction

Soil-transmitted helminth (STH) infections remain a persistent public health concern in sub-Saharan Africa (SSA). The principal STHs infecting humans include *Ascaris lumbricoides, Trichuris trichiura* and hookworms *Ancylostoma duodenale, Necator americanus* and *Ancylostoma ceylanicum*.^1^ These infections are associated with substantial morbidity, particularly in children and pregnant women, leading to malnutrition, iron-deficiency anaemia, impaired cognitive and physical development and adverse pregnancy outcomes.^1^  *Strongyloides stercoralis*, although not routinely targeted by STH control programs, is increasingly recognised as an STH of public health importance due to its capacity for lifelong infection via autoinfection and the risk of severe disease in immunocompromised individuals.^2^ Zoonotic STHs of non-human primates (NHPs), including *Strongyloides fuelleborni* and *Trichuris incognita*, have also been reported in human populations in parts of SSA.^3,4^ Emerging evidence suggests that *A. lumbricoides* and the zoonotic porcine parasite *Ascaris suum* may represent a species complex, which can complicate species-level attribution of human *Ascaris* infections in the absence of molecular sequencing.^5^

STH infections are primarily acquired through contact with soil contaminated by human faeces containing infective eggs or larvae.^1^ Globally, an estimated 1.5 billion people are infected, with the highest burdens occurring in tropical and subtropical regions characterised by persistent poverty, inadequate sanitation and limited public health infrastructure.^1^ Control strategies have traditionally relied on periodic mass drug administration (MDA) with benzimidazole anthelmintics (albendazole or mebendazole) delivered to preschool- and school-aged children, complemented by water, sanitation and hygiene (WASH) interventions aimed at reducing transmission.^6^ Ivermectin is currently considered the most effective treatment for *Strongyloides* infections.^7^ The World Health Organisation’s (WHO) 2030 roadmap for neglected tropical diseases calls for the expansion of STH control to all at-risk populations and the establishment of dedicated programs for strongyloidiasis.^6,7^ Robust, region-specific epidemiological surveillance is essential to guide the design and implementation of targeted control strategies.

Gabon, located in Central Africa, has ecological and socio-economic conditions conducive to STH transmission, including a tropical climate, high rainfall and ecosystems dominated by rainforest and savannah.^8,9^ Although Gabon has one of the highest per capita incomes in SSA, significant socio-economic disparities persist. Many communities experience high levels of poverty, inadequate sanitation, limited access to clean water and underresourced healthcare services.^8^ Environmental changes driven by logging and infrastructure development may have increased human–wildlife interactions, elevating the risk of zoonotic STH transmission from NHPs.^9^

A 2018 geospatial meta-analysis of STH infections in children across SSA identified Gabon as one of the highest transmission hotspots, with a prevalence >50% in some regions, including Ngounié Province.^10^ Over the past decade, seven faecal surveys have been conducted in Gabon, reporting STH prevalences ranging from 15 to 56% in children and 7 to 38% in adults.^11–17^ All studies employed microscopy-based diagnostics, including Kato–Katz,^12–17^ direct smear^11^ and the merthiolate–iodine–formaldehyde concentration technique;^11^ some studies also used coproculture to detect *Strongyloides* larvae.^11,13–16^ The reported prevalence of *S. stercoralis* ranged from 1 to 11%.^11,13–16^

Microscopy has limited capacity to differentiate hookworm species, and identification of *S. fuelleborni* can be complicated by morphological similarity to embryonated hookworm eggs, as well as the indistinguishability of hatched larvae from those of *S. stercoralis*.^18–20^ Molecular diagnostics such as quantitative polymerase chain reaction (qPCR) have demonstrated higher sensitivity than microscopy for detecting STH infections and enable species-level identification of human hookworms.^18,19^

Gabon initiated school-based MDA with albendazole in 2021, achieving 46% coverage in 2022; however, implementation areas were not documented and program activities were curtailed in 2023 due to funding constraints.^21^ Although multiple school-based STH surveys have been conducted in the country,^12,13^ community-level epidemiological data remain limited and no molecular surveillance of STHs has been undertaken to date. Community-based surveillance using sensitive molecular diagnostics is essential to define region-specific STH epidemiology and support the WHO’s call for expanded control targeting all at-risk populations. Identifying sociodemographic risk factors is a key component of such surveillance, as it enables prioritisation of high-risk groups and the development of targeted interventions. Accordingly, we conducted a community-based survey in Gabon using both microscopy and qPCR to determine the prevalence of human STH infections and to assess associated sociodemographic risk factors.

## Methods

### Study area and sampling

**Figure 1:**
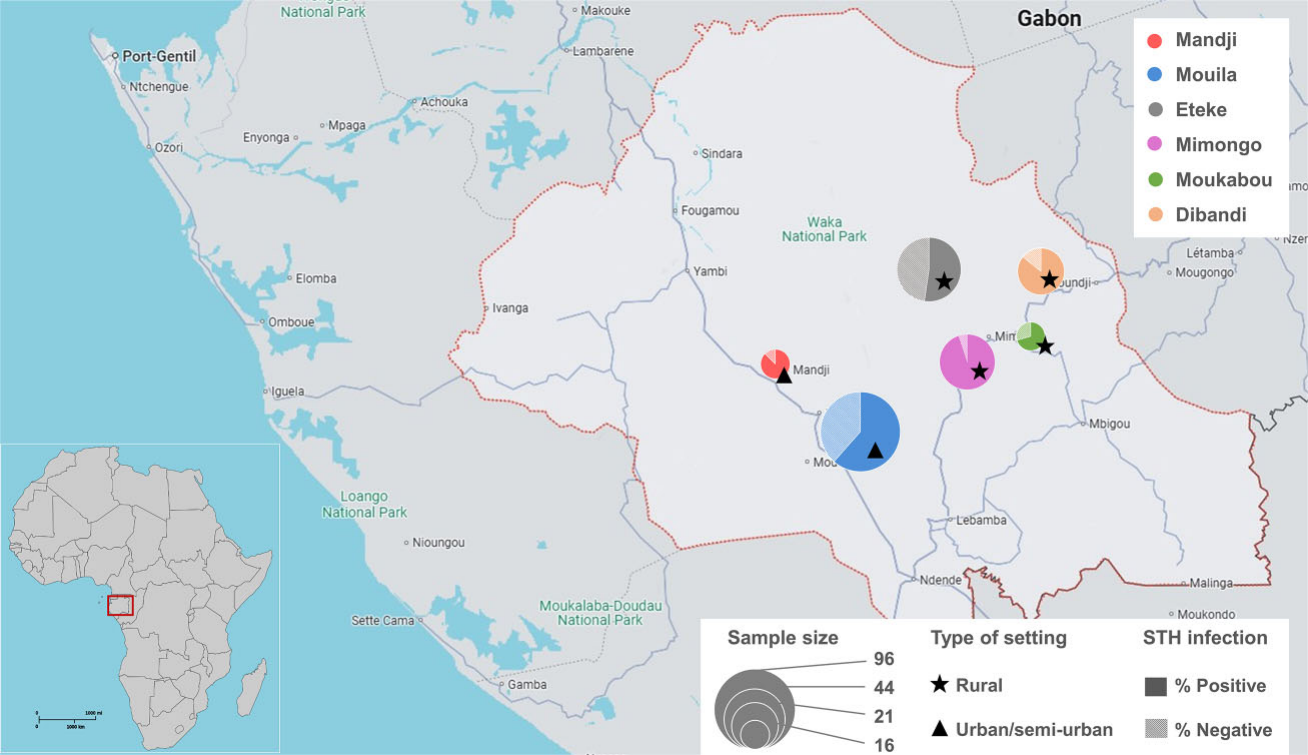
Sampling locations in Ngounié Province, Gabon. Pie charts represent villages where stool samples were collected, with chart size proportional to the number of samples obtained at each site. Colours distinguish individual villages. Each site is marked with either a star to indicate a rural setting or a triangle to indicate an urban/semi-urban setting. Within each pie chart, solid segments indicate the percentage of individuals testing positive for STHs, while patterned segments indicate the percentage testing negative.

A cross-sectional survey was conducted in Ngounié Province, Gabon, during the dry season in July 2023. Ngounié is located in south-central Gabon within a tropical savannah climate zone and is characterised by a predominantly rural, forested landscape. In 2020, the province had an estimated population of 121 200 and a population density of approximately 8.1 persons per km^2^.^22^ Participants were recruited using convenience sampling from six communities, comprising four rural villages (Mimongo, Moukabou, Eteke and Dibandi) and two urban or semi-urban centres (Mouila and Mandji) (Figure 1). Study sites were selected based on accessibility and logistical feasibility, with the aim of capturing a range of rural–urban settings within the province. All residents present in the communities at the time of the survey were eligible to participate, with no age restrictions, except for individuals unable or unwilling to provide a stool sample. Based on the most recently reported STH prevalence of 21% in neighbouring Moyen-Ogooué Province,^16^ we estimated that a minimum of 218 samples was required to estimate prevalence with 95% confidence and a 5% margin of error, assuming the approximate sensitivity (92%) and specificity (98%) of the diagnostic methods used (microscopy and qPCR). To account for potential sample loss, insufficient stool volume or DNA extraction failure, recruitment continued beyond this minimum, resulting in the collection of 233 stool samples, all of which were included in the final analysis.

Participants were approached in person by the researchers. Each participant was provided with a sterile specimen container and instructions for stool collection. A single stool sample was collected from each participant and returned promptly to the research team. Sociodemographic data (age, sex and address) were recorded. Upon receipt, each sample was split, with approximately 1 g preserved in 10% formalin and 500 mg in 70% ethanol, both stored at room temperature. Due to the small sample amount, six samples were preserved only in formalin and seven only in ethanol. All samples were shipped to James Cook University, Australia, for laboratory analysis.

All participants provided written informed consent for their samples to be processed and data published. For those <18 y of age, consent was obtained from a legal guardian and assent was provided by the participant. The study was approved by the Ministry of Health and Social Affairs Scientific and Ethics Committee of the CHU Mère-Enfant Fondation Jeanne Ebori, Gabon (015/MSAS/CHUMEFJE/DG/DAM/CS) and the Human Research Ethics Committee of James Cook University, Australia (H9547).

### Laboratory analysis

Formalin-preserved stool samples (n=226) were analysed by microscopy using the formalin–ethyl acetate sedimentation (FES) method. Approximately 1 g of stool was processed per sample, following previously described procedures.^23^ Two 22 × 22 mm coverslips were prepared from each concentrated sample and examined independently by two trained parasitologists at 100× and 400× magnifications. A sample was classified as positive if either examiner detected STH eggs or larvae, and discrepant results were resolved through joint re-examination.

Ethanol-preserved stool samples (n=227) underwent DNA extraction using the PowerSoil DNA kit (Qiagen, Hilden, Germany). Approximately 200 mg of each sample was centrifuged at 8000 *g* for 60 s to remove residual ethanol, followed by an overnight wash in 1 ml of Milli-Q water. Samples were then centrifuged again (8000 *g* for 60 s) and the supernatant was discarded. Bead-beating lysis was performed for 1 min using a FastPrep-24 5G Instrument (MP Biomedicals, Solon, OH, USA). All subsequent steps followed the manufacturer’s protocol. DNA was eluted in 100 μl of solution C6 and stored at −20°C until qPCR analysis.

Stool DNA extracts (n=227) were analysed using the Allplex GI-Helminth(I) Assay (Seegene, Seoul, South Korea), a commercial multiplex qPCR assay capable of detecting eight helminths and one protozoan.^24^ This study reports results for *Ascaris* spp., *Ancylostoma* spp., *Necator americanus, Trichuris* spp. and *Strongyloides* spp. A separate qPCR assay targeting the human cytochrome b (cytb) gene was used to assess DNA extraction success and PCR inhibition. Each qPCR run included a no-template control and positive controls containing parasite genomic DNA. All samples were tested in duplicate and a sample was considered positive if both replicates produced a valid amplification curve crossing the assay-defined cycle threshold. Runs were repeated if discrepancies were observed between replicates, control reactions failed or inhibition was detected.

### Statistical analysis

Data were entered into a password-protected spreadsheet (Excel, Microsoft, Redmond, WA, USA), cross-checked against the original records for accuracy and cleaned prior to analysis. Power analysis was conducted using Epitool (https://epitools.ausvet.com.au/) and all statistical analyses were performed in SPSS version 29.0 (IBM, Armonk, NY, USA), with a significance level of p<0.05.

The prevalence of STH infections was determined separately by microscopy and qPCR, as well as using a composite infection status in which samples were considered positive if either method detected the infection. Prevalence estimates were stratified by age, sex and locality and reported with 95% confidence intervals (CIs). Diagnostic agreement between microscopy and qPCR was assessed using Cohen’s κ, interpreted as follows: ≤0.00 (poor), 0.01–0.20 (slight), 0.21–0.40 (fair), 0.41–0.60 (moderate), 0.61–0.80 (substantial) and 0.81–1.00 (perfect). Multivariate logistic regression was used to examine associations between STH infection and sociodemographic variables, including age (continuous or categorical), sex (female vs male) and locality (rural vs urban/semi-urban).

## Results

### Participant demographics

Of the 233 participants enrolled, 52% were from rural areas and 48% from urban/semi-urban settings. Age and sex data were available for 180 participants due to missing demographic information for 53 individuals at the Mouila study site. Among these, 78 were male and 102 were female. Participant ages ranged from 2 to 89 y, with a mean age of 27 y (standard deviation 20.4).

### STH overall prevalence and coinfections

**Figure 2:**
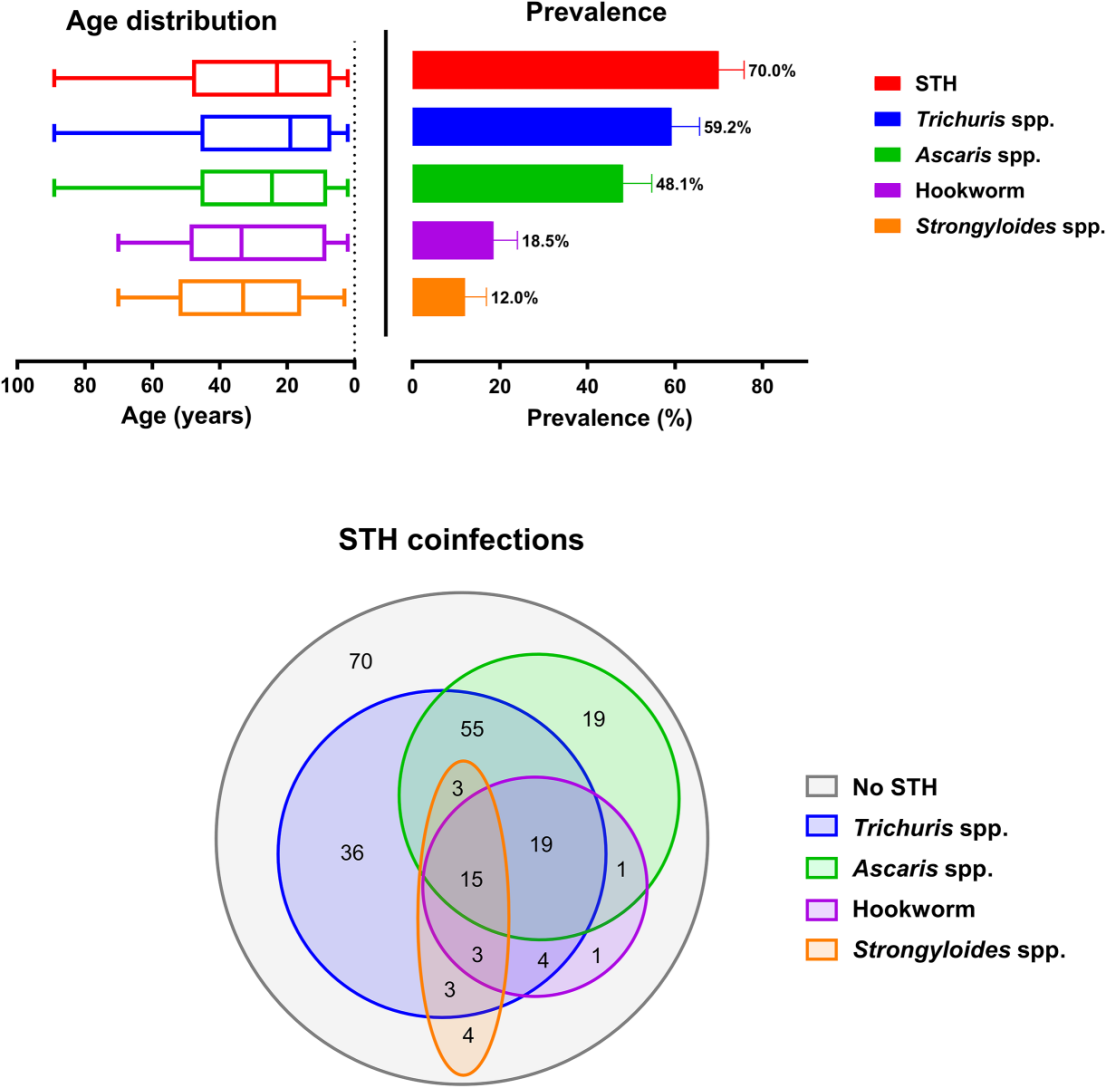
Overall prevalence and age distribution of STH infections in Gabonese humans and the number of individuals with single and multiple STH infections.

The overall prevalence of STH infections in the study cohort (n=233), based on a composite of microscopy and qPCR results, was 70.0% (163/233 [95% CI 63.6 to 75.8]). The most frequently detected STH was *Trichuris* spp. (138/233 [59.2%]; 95% CI 52.6 to 65.6), followed by *Ascaris* spp. (112/233 [48.1%]; 95% CI 41.5 to 54.7), hookworms (43/233 [18.5%]; 95% CI 13.7 to 24.0) and *Strongyloides* spp. (28/233 [12.0%]; 95% CI 8.1 to 16.9). Single STH infections were identified in 25.8% (60/233 [95% CI 20.3 to 31.9%]) of participants, whereas coinfections were detected in 44.2% (103/233 [95% CI 37.7 to 50.8]) (Figure 2).

STH infections were more prevalent among females, adults 18–40 y of age and individuals residing in rural areas. In contrast, *Trichuris* spp. was more frequently detected in children (≤17 y) than in adults. Prevalence also varied across communities, with higher rates observed in Mimongo and Mandii compared with the other study sites (Table 1).

**Table 1:** Prevalence of STH infections stratified by sex, age group, community and economic setting (N=233).

		Prevalence, % (95% CI)^a^				
Characteristics	n (%)	STH	*Ascaris* spp.	*Trichuris* spp.	Hookworms	*Strongyloides* spp.
Sex						
Male	78 (33.5)	66.7 (55.1 to 76.9)	46.2 (34.8 to 57.8)	55.1 (43.4 to 66.4)	20.5 (12.2 to 31.2)	19.2 (11.2 to 29.7)
Female	102 (43.8)	73.5 (63.9 to 81.8)	47.1 (37.1 to 57.2)	63.7 (53.6 to 73.0)	15.7 (9.2 to 24.2)	6.9 (2.8 to 13.6)
Unreported	53 (22.7)	67.9 (53.7 to 80.1)	52.8 (38.6 to 66.7)	56.6 (42.3 to 70.2)	20.8 (10.8 to 34.1)	11.3 (4.3 to 23.0)
Age group (years)						
0–17	80 (34.3)	71.3 (60.1 to 80.8)	46.3 (35.0 to 57.8)	65.0 (53.5 to 75.3)	12.5 (6.2 to 21.8)	6.3 (2.1 to 14.0)
18–40	47 (20.2)	72.3 (57.4 to 84.4)	51.1 (36.1 to 65.9)	55.3 (40.1 to 69.8)	23.4 (12.3 to 38.0)	21.3 (10.7 to 35.7)
≥41	53 (22.7)	67.9 (53.7 to 80.1)	43.4 (29.8 to 57.7)	56.6 (42.3 to 70.2)	20.8 (10.8 to 34.1)	13.2 (5.5 to 25.3)
Unreported	53 (22.7)	67.9 (53.7 to 80.1)	52.8 (38.6 to 66.7)	56.6 (42.3 to 70.2)	20.8 (10.8 to 34.1)	11.3 (4.3 to 23.0)
Economic setting						
Urban/semi-urban	112 (48.1)	65.2 (55.6 to 73.9)	42.0 (32.7 to 51.7)	51.8 (42.2 to 61.3)	17.9 (11.3 to 26.2)	8.0 (3.7 to 14.7)
Rural	121 (51.9)	74.4 (65.7 to 81.9)	53.7 (44.4 to 62.8)	66.1 (57.0 to 74.5)	19.0 (12.5 to 27.1)	15.7 (9.7 to 23.4)
Community						
Mimongo	39 (16.7)	94.9 (82.7 to99.4)	84.6 (69.5 to 94.1)	89.7 (75.8 to 97.1)	20.5 (9.30 to 36.5)	18.0 (7.5 to 33.5)
Moukabou	17 (7.3)	70.6 (44.0 to 89.7)	29.4 (10.3 to 56.0)	58.8 (32.9 to 81.6)	11.8 (1.5 to 36.4)	0
Eteke	44 (18.9)	52.3 (36.7 to 67.5)	27.3 (15.0 to 42.8)	43.2 (28.4 to 59.0)	4.6 (0.6 to 15.5)	4.6 (0.6 to 15.5)
Dibandi	21 (9.0)	85.7 (63.7 to 97.0)	71.4 (47.8 to 88.7)	76.2 (52.8 to 91.8)	52.4 (29.8 to 74.3)	47.6 (25.7 to 70.2)
Mouila	96 (41.2)	61.5 (51.0 to 71.2)	39.6 (29.8 to 50.1)	47.9 (37.6 to 58.4)	12.5 (6.6 to 20.8)	8.3 (3.7 to 15.8)
Mandji	16 (6.9)	87.5 (61.7 to 98.5)	56.3 (29.9 to 80.3)	75.0 (47.6 to 92.7)	50.0 (24.7 to 75.4)	6.3 (0.2 to 30.2)

^a^Prevalence based on microscopy and qPCR.

### Prevalence of infections by microscopy vs qPCR

**Figure 3:**
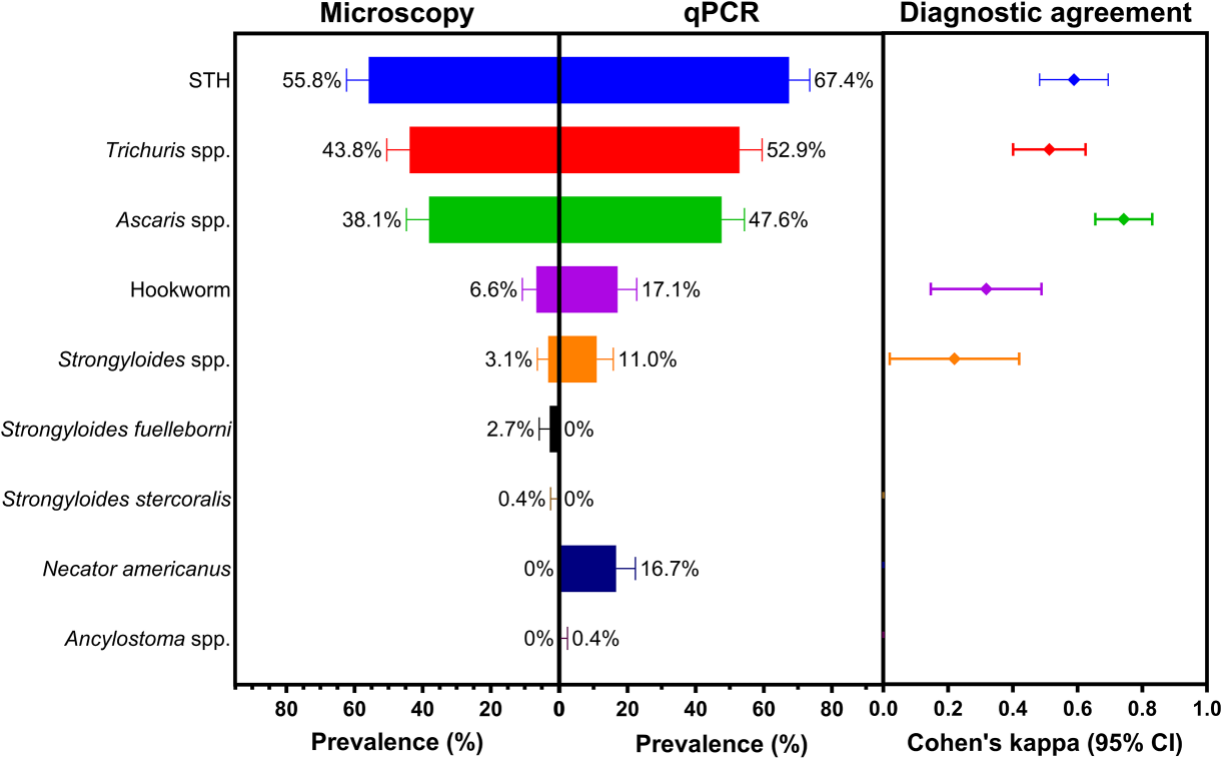
Prevalence of STHs in Gabonese humans by microscopy (n=226) vs by qPCR (n=227) and their diagnostic agreement (n=220).

Of the 226 samples analysed by microscopy, 55.8% (126/226 [95% CI 49.0 to 62.3]) tested positive for at least one STH. Detected STHs included *Trichuris* spp. (43.8% [99/226]; 95% CI 37.2 to 50.5), *Ascaris* spp. (38.1% [86/226]; 95% CI 31.7 to 44.7), hookworms (6.6% [15/226]; 95% CI 3.8 to 10.7), *S. fuelleborni* (2.7% [6/226]; 95% CI 1.0 to 5.7) and *S. stercoralis* (0.4% [1/226]; 95% CI 0 to 2.4) (Figure 3).

All 227 ethanol-preserved samples yielded amplifiable human DNA. qPCR analysis of these DNA extracts revealed a higher STH prevalence of 67.4% (153/227 [95% CI 60.9 to 73.5]), with increased detection of *Trichuris* spp. (52.9% [120/227]; 95% CI 46.2 to 59.5), *Ascaris* spp. (47.6% [108/227]; 95% CI 41.0 to 54.3) and hookworms (17.1% [39/227]; 95% CI 12.5 to 22.7). Among hookworm-positive samples, *N. americanus* predominated (16.7% [38/227]; 95% CI 12.1 to 22.3), while *Ancylostoma* spp. were detected in a single sample (0.4% [1/227]; 95% CI 0 to 2.4). *Strongyloides* spp. were identified in 11.0% (25/227 [95% CI 7.3 to 15.8]) of samples, including three of the six *S. fuelleborni* and the single *S. stercoralis* infections detected by microscopy (Figure 3).

Diagnostic agreement (n=220) between microscopy and qPCR was substantial for *Ascaris* spp. (κ=0.74 [95% CI 0.66 to 0.83]), moderate for *Trichuris* spp. (κ=0.51 [95% CI 0.40 to 0.63]) and fair for hookworms (κ=0.32 [95% CI 0.15 to 0.4]9) and *Strongyloides* spp. (κ=0.22 [95% CI 0.02 to 0.42]) (Figure 3; Supplementary Table).

### Risk profiling of STH infections

Rural residence was identified as an independent predictor of both *Ascaris* spp. and *Trichuris* spp. infections. Compared with individuals from urban or semi-urban areas, those living in rural settings had significantly higher odds of *Ascaris* spp. infection (model 1: adjusted odds ratio [AOR] 2.48 [95% CI 1.27 to 4.84], p=0.008; model 2: AOR 2.50 [95% CI 1.29 to 4.86], p=0.007) and *Trichuris* spp. infection (model 1: AOR 2.62 [95% CI 1.34 to 5.12], p=0.005; model 2: AOR 2.45 [95% CI 1.27 to 4.73], p=0.008). In the larger cohort assessed in model 3, the association between rural residence and *Trichuris* spp. infection remained statistically significant (AOR 1.82 [95% CI 1.07 to 3.08], p=0.027) (Table 2).

**Table 2:** Logistic regression modelling for the risk of STH infections in Gabonese humans.

	STH		*Ascaris* spp.		*Trichuris* spp.		Hookworms		*Strongyloides* spp.	
Characteristics	AOR (95% CI)	p-Value	AOR (95% CI)	p-Value	AOR (95% CI)	p-Value	AOR (95% CI)	p-Value	AOR (95% CI)	p-Value
**Model 1 (n=180)**										
Sex										
Female^a^	–	–	–	–	–	–	–	–	–	–
Male	0.64 (0.32 to 1.25)	0.190	0.85 (0.46 to 1.58)	0.611	0.54 (0.29 to 1.04)	0.064	1.56 (0.70 to 3.47)	0.278	3.83 (1.39 to 10.55)	0.010
Age group (years)										
0–17^a^	–	–	–	–	–	–	–	–	–	–
18–40	0.87 (0.38 to 2.00)	0.740	1.02 (0.48 to 2.17)	0.952	0.49 (0.22 to 1.08)	0.077	2.29 (0.86 to 6.11)	0.096	4.90 (1.45 to 16.59)	0.011
≥41	0.78 (0.36 to 1.70)	0.535	0.85 (0.42 to 1.75)	0.667	0.61 (0.29 to 1.29)	0.199	1.96 (0.76 to 5.08)	0.165	2.74 (0.79 to 9.52)	0.113
Economic setting										
Urban/semi-urban^a^	–	–	–	–	–	–	–	–	–	–
Rural	1.87 (0.94 to 3.74)	0.076	2.48 (1.27 to 4.84)	0.008	2.62 (1.34 to 5.12)	0.005	1.11 (0.46 to 2.67)	0.810	2.34 (0.63 to 8.68)	0.204
Intercept	2.18 (1.09 to 4.38)	0.028	0.53 (0.27 to 1.03)	0.060	1.46 (0.75 to 2.85)	0.264	0.10 (0.04 to 0.27)	<0.001	0.02 (0 to 0.07)	<0.001
**Model 2 (n=180)**										
Sex										
Female^a^	–	–	–	–	–	–	–	–	–	–
Male	0.66 (0.34 to 1.28)	0.217	0.86 (0.46 to 1.58)	0.620	0.59 (0.31 to 1.11)	0.100	1.44 (0.66 to 3.16)	0.361	3.32 (1.24 to 8.92)	0.017
Age (years) as a continuous variable	0.99 (0.98 to 1.02)	0.938	1.00 (0.98 to 1.02)	0.976	0.99 (0.98 to 1.01)	0.302	1.01 (0.99 to 1.03)	0.212	1.02 (0.99 to 1.04)	0.073
Economic setting										
Urban/semi-urban^a^	–	–	–	–	–	–	–	–	–	–
Rural	1.85 (0.93 to 3.66)	0.079	2.50 (1.29 to 4.86)	0.007	2.45 (1.27 to 4.73)	0.008	1.18 (0.49 to 2.78)	0.710	2.77 (0.76 to 10.03)	0.122
Intercept	1.98 (0.96 to 4.07)	0.064	0.50 (0.25 to 1.01)	0.055	1.31 (0.66 to 2.61)	0.437	0.12 (0.05 to 0.30)	<0.001	0.02 (0 to 0.08)	<0.001
**Model 3 (n=233)**										
Economic setting										
Urban/semi-urban^a^	–	–	–	–	–	–	–	–	–	–
Rural	1.55 (0.88 to 2.73)	0.127	1.61 (0.96 to 2.70)	0.073	1.82 (1.07 to 3.08)	0.027	1.08 (0.56 to 2.10)	0.821	2.13 (0.92 to 4.93)	0.077

^a^Reference category.

Sex was not significantly associated with infection risk for most STHs. However, male participants had greater than threefold increased odds of *Strongyloides* spp. infection compared with females (model 1: AOR 3.83 [95% CI 1.39 to 10.55]; p=0.010; model 2: AOR 3.32 [95% CI 1.24 to 8.92], p=0.017) (Table 2).

Age-related differences were most pronounced for *Strongyloides* spp. Adults 18–40 y of age had nearly fivefold higher odds of infection compared with participants <18 y of age (AOR 4.90 [95% CI 1.45 to 16.59], p=0.011). No significant associations with age were observed for other STHs, although a non-significant trend towards reduced odds of *Trichuris* spp. infection in adults was noted (AOR range 0.49–0.61 in model 1). When age was modelled as a continuous variable, no significant associations with infection status were found for any STH (Table 2).

## Discussion

This study provides the first molecular surveillance data on human STH infections in Gabon. The findings indicate that STH infections remain endemic in certain rural and urban/semi-urban communities of south-central Gabon. As of 2023, Gabon remains one of the few counties in Africa without sustained nationwide MDA coverage for STHs and without ivermectin-based MDA for onchocerciasis or lymphatic filariasis, the latter partly due to challenges associated with *Loa loa* co-endemicity.^21^ This community-based survey provides sensitive, region-specific epidemiological data that can inform the future implementation of targeted control programs.

In this study, the prevalence of STH infections detected by FES microscopy (55.8%) was higher than that reported in previous surveys from central western Gabon (8–44%),^11,14,16^ but lower than the 65% reported by Mouandza et al.^15^ in southern Gabon. The inclusion of qPCR increased overall detection by 12%, reflecting the superior sensitivity of molecular diagnostics.^18,19^ The combined use of qPCR and microscopy increased detection of hookworms by 11% and *Strongyloides* spp. by 8% compared with microscopy alone. Similar findings have been reported from other endemic settings.^25,26^ These results suggest that integrating molecular diagnostics with traditional microscopy can enhance case detection and improve the sensitivity of epidemiological surveillance.

However, several hookworm infections detected by microscopy were not identified by the Allplex GI-Helminth(I) qPCR assay. Subsequent *cox1* genotyping in a separate study^27^ identified four of these cases as *Necator gorillae* Noda & Yamada, 1964, a zoonotic parasite of NHPs. This finding raises the possibility that commonly used qPCR assays, including Allplex GI-Helminth(I), may have limited ability to detect zoonotic *Necator* spp. or to differentiate them from *N. americanus*. Similar concerns apply to *T. trichiura* and *A. lumbricoides* detection, where cross-amplification with zoonotic *Trichuris* spp. and *Ascaris* spp. cannot be excluded.^3,5^ While this study detected *Trichuris* and *Ascaris* infections, species-level identification was not possible with the assays used. Future surveillance in SSA may therefore benefit from sequencing-based approaches to more precisely determine the species of these infections. Of particular concern is the NHP whipworm *T. incognita*, recently reported in human populations in West Africa and shown to have reduced responsiveness to combined albendazole–ivermectin therapy.^3^

*Trichuris* spp. was the most prevalent STH, followed by *Ascaris* spp. and hookworms, consistent with regional modelling data for Central Africa^10^ and previous reports from Gabonese communities.^11–15^ Rural residence was identified as an independent risk factor for infection with *Trichuris* spp. and *Ascaris* spp., in line with findings from endemic regions of South America^28^ and Southeast Asia.^29^ This likely reflects increased environmental exposure in rural settings, including inadequate sanitation and limited hygiene infrastructure that promote faecal contamination of soil.^8^ A recent community-based survey in southeastern Gabon reported that only 10% of households had access to improved sanitation facilities, while 80% of participants practiced open defecation.^17^ Future studies could explore specific rural environmental, WASH and socio-economic factors that mediate the relationship between rural residence and STH infection risk in the studied communities.

The majority of hookworm infections were attributable to *N. americanus*, consistent with reports from the neighbouring country, Cameroon.^30^ The prevalence of *Strongyloides* spp. exceeded most previous reports from Gabon based on coproculture,^11,13,15,16^ but was comparable to the 11% reported in Moyen-Ogooué Province by Edoa et al.^14^ Differences in diagnostic sensitivity may partly explain this variation, although some coproculture methods, such as agar plate culture, have demonstrated higher sensitivity than qPCR for *Strongyloides* spp. detection.^18^ In addition, the absence of targeted control for strongyloidiasis in Gabon may contribute to sustained transmission. In contrast to countries where ivermectin-based MDA for onchocerciasis or lymphatic filariasis has led to collateral reductions in *Strongyloides* prevalence,^7^ no such programs are currently implemented in Gabon.

The detection of *S. fuelleborni* in 2.7% of participants adds to limited contemporary data on this parasite in human populations. The presence of *S. fuelleborni* eggs in stool could reflect zoonotic transmission from NHPs, locally established transmission cycles in humans or transient passage of ingested eggs. Few large-scale surveys have assessed *S. fuelleborni* in human populations in Africa since the 1980s, although earlier studies reported a prevalence of 13% (606/4577) across tropical African regions^31^ and 10–31% in clinical specimens from Zambia.^32^ More recent molecular surveys have identified a high prevalence of human infections in Asia and the Pacific region.^33,34^ Together, these findings suggest that *S. fuelleborni* may be underrecognised in routine diagnostics, potentially due to misidentification as embryonated hookworm eggs during microscopy.^20^ Although a *S. fuelleborni*–specific qPCR assay with improved sensitivity has recently been developed,^35^ it has yet to be deployed in large-scale epidemiological studies. In regions of Central Africa undergoing ecological disruption and increasing human–NHP contact, targeted molecular surveillance, including genotyping of isolates from humans and sympatric NHPs, could help clarify transmission pathways and better define the distribution and public health relevance of this neglected STH.

Several limitations of this study should be acknowledged. First, the study relied on convenience sampling and opportunistic recruitment of available community members. Participants were not randomly selected and the overall participation rate is unknown. The sociodemographic representativeness of the sample could not be assessed because comparable community-level data were unavailable. These factors may have introduced selection bias and limit the generalisability of the findings to the wider population of Ngounié Province. Second, demographic data (age and sex) were missing for 23% of participants, substantially reducing the effective sample size for multivariate analyses and potentially biasing risk factor estimates, particularly for *Strongyloides* spp., where case numbers were low. Third, 6% of samples (13/233) were tested by only one diagnostic method (microscopy or qPCR), limiting the number available for direct diagnostic comparison. Fourth, FES microscopy has low sensitivity for detecting *S. stercoralis* larvae in stool, for which reliable detection depends primarily on qPCR. Finally, this study did not collect environmental, WASH or broader socio-economic data, restricting the ability to assess key determinants of STH transmission.

## Conclusions

This study represents the first molecular surveillance of human STH infections in Gabon, confirming the continued endemicity of *Trichuris* spp., *Ascaris* spp., *N. americanus* and *Strongyloides* spp. infections in certain south-central communities. Rural residency was identified as a key risk factor for *Trichuris* spp. and *Ascaris* spp. infections, highlighting the need for future studies to investigate the environmental, WASH and socio-economic factors underlying this association. The detection of the rare zoonotic NHP parasite *S. fuelleborni* indicates the need for larger-scale surveys to clarify its epidemiology in Gabon and across Central Africa. Our findings also highlight limitations of FES microscopy and the Allplex GI-Helminth(I) qPCR assay in detecting certain STH species, reinforcing the importance of incorporating species-specific molecular diagnostics into future surveillance efforts.

## Supplementary Material

trag038_Supplemental_File

## Data Availability

The datasets used and/or analysed during the current study are available from the corresponding author upon reasonable request.
