# Supplementary material for: Surveillance and sociodemographic risk profiling of human soil-transmitted helminth infections in Gabon, Central Africa
Source: Trans R Soc Trop Med Hyg. 2026 Apr 29;120(7):771–80. doi: 10.1093/trstmh/trag038 (PMC13339095; doi:10.1093/trstmh/trag038)
Supplement: trag038_Supplemental_File [file trag038_supplemental_file.docx]

**Supplementary Table.** Diagnostic agreement between microscopy and qPCR for STH detection (n=220).

|  | Cohen’s kappa (95% CI) | p value |
| --- | --- | --- |
| STH | 0.589 (0.483, 0.695) | <0.01 |
| *Ascaris* spp. | 0.743 (0.655, 0.831) | <0.01 |
| *Trichuris* spp. | 0.513 (0.401, 0.625) | <0.01 |
| Hookworm | 0.318 (0.147, 0.489) | <0.01 |
| *Strongyloides* spp. | 0.220 (0.02, 0.420) | <0.01 |
